# Vaccination and Malaria Prevention among International Travelers Departing from Athens International Airport to African Destinations

**DOI:** 10.1155/2014/563030

**Published:** 2014-03-02

**Authors:** Androula Pavli, Athina Spilioti, Paraskevi Smeti, Stavros Patrinos, Helena C. Maltezou

**Affiliations:** Department for Interventions in Health Care Facilities, Hellenic Center for Disease Control and Prevention, 3-5 Agrafon Street, 15123 Athens, Greece

## Abstract

*Background*. International travel to Africa has grown dramatically over the last decade along with an increasing need to understand the health issues for travelers. The current survey aimed to assess vaccination and malaria prevention of travelers visiting Africa. *Methods*. A questionnaire-based survey was conducted from of November 1, 2011 to of April 30, 2013 at Athens International Airport. *Results*. A total of 360 travelers were studied; 68% were men. Their mean age was 39.9 years. Previous travel to tropical countries was reported by 71.9% of them. Most frequent destination was sub-Saharan Africa (60%). Most of them traveled for ≥1 month (62%). The main reason for travel was work (39.7%). Only 47% sought pretravel consultation. Hepatitis A, typhoid, and meningococcal vaccines were administered to 49.8%, 28%, and 26.6%, respectively, and malaria chemoprophylaxis to 66.8% of those who visited sub-Saharan Africa. A history of previous travel to a tropical country, elementary level of education, and traveling for visiting friends and relatives, and for short duration were significant determinants for not pursuing pretravel consultation. *Conclusions*. The current survey revealed important inadequacies in vaccine and malaria prophylaxis of travelers departing to Africa. Educational tools should be developed in order to improve awareness of travelers to risk destinations.

## 1. Introduction

International travel has increased in the last six decades worldwide with the highest increase noted in tropical and subtropical areas. International tourist arrivals grew by 4% in 2012 and for the first time in history exceeded one billion. In 2012, a 6% increase was recorded for Africa, equivalent to 3 million more travelers, reaching 50 million for the first time ever [1]. Travelers to tropical and subtropical countries can be exposed to various infectious diseases and may facilitate their importation into countries where these diseases are not endemic [2, 3]. Approximately 8% of travelers to developing countries require medical care during or after travel; the main diagnoses include acute diarrhoea, malaria, and dengue fever [3]. Sub-Saharan Africa was one of the most common regions (26.7%) where illnesses were acquired, as recorded by the GeoSentinel surveillance network [4].

The importation of vaccine-preventable diseases has been recognized as an important travel-related problem [5]. Vaccine preventable diseases (VPDs) accounted for 1.5% of ill returned travelers, with enteric fever, acute viral hepatitis, and influenza as the most common diagnoses [5]. Malaria is one of the most important travel associated diseases due to its widespread geographic distribution and its potential fatal outcome if untreated, especially in nonimmune individuals. Malaria diagnosis accounted for 29% of those with fever and disproportionally in travelers returning from Africa [4]; according to the global data base, the majority of the infections were acquired in sub-Saharan Africa and 60% of them were due to *Plasmodium falciparum* [6].

As a result of growing international travel, health consultants are increasingly likely to be consulted for advice before travel or by ill returned travelers. Previous studies have revealed that a large proportion of travelers do not seek pretravel advice [7–12]. The current study aimed to assess pretravel consultation seeking practices and provision of vaccinations and malaria prophylaxis among international travelers departing from Athens International Airport and visiting African countries.

## 2. Materials and Methods

A prospective questionnaire-based study was conducted from November 1, 2011 to April 30, 2013. Data collection was carried out 2 days weekly. Travelers leaving from the departure gates of Athens International Airport were invited to participate in the survey. Selection criteria were being a Greek resident, ≥19 years old, and traveling to Africa. Travelers participated on a voluntary basis; no incentive was provided. Permission was given by the International airport Authority, the airlines flying to the above destinations, and the Hellenic Center for Disease Control and Prevention (HCDCP).

Data were collected using a standardized anonymous questionnaire specifically designed in Greek language. It was administered by 2 trained interviewers and lasted about 10 minutes. Data included information about demographic and travel characteristics, and pretravel consultation (vaccination, malaria prophylaxis, general preventive measures, and source of pretravel consultation). Up to 5 travelers were interviewed per day, depending on the selection criteria.

High risk areas and those with moderate-high prevalence of malaria were defined according to published sources [6, 13]. The definition of high risk travelers with regard to exposure to malaria in endemic areas was based also on travel (area and place of stay, duration and purpose of travel, and activities) and travelers' characteristics (age, behaviour, and medical history). Adequate vaccine and malaria prophylaxis is considered according to the national Hellenic Centre for Disease Control and Prevention guidelines, which are in accordance with the World Health Organization and US Centers for Disease Control and Prevention guidelines [14–16]. Urban accommodation was defined as cities with population of 5,000 people or more, whereas rural accommodation was defined as villages with up to population of 5,000 or staying in the countryside (Hellenic Statistics Authority: personal communication). Short-term travel was defined as a trip of <1 month duration, while long-term travel was defined as a trip of ≥1 month. An organized trip was defined as a guided, package trip, mainly to popular tourist destinations. Outdoor activities include adventure sport, backpacking, hiking, and remote expedition. Travelers VFRs were classified as those immigrants to Greece who return to their homeland, a lower income country, to visit friends or relatives according to a definition outlined by the US Centers for Disease Control and Prevention (CDC) [17].

Chi-squared test and Fisher's exact test were used for comparison between categorical variables. The standard multiple logistic regression was conducted (if *P* value < 0.15 in univariate analysis) to examine the relation between pretravel preparation and independent factors. Multivariate analysis was used in order to identify factors influencing the attitude to pretravel consultation. Odds ratios (ORs) and their 95% confidence intervals (CIs) were calculated. *P* values of 0.05 or less were considered statistically significant. Unanswered items were excluded from the analysis. Analysis was performed using STATA v12.1 software.

## 3. Results

A total of 360 travelers participated in the survey (98% participation rate); 60%, 35%, and 5% were traveling to sub-Saharan Africa, North Africa, and Southern Africa region (based on United Nations' classification), respectively; more than one quarter of them visited for the first time a tropical or subtropical country. The number of travelers to African destinations was approximately stable throughout the study period. The five most common African countries were Egypt (35%), Ethiopia (13.3%), Kenya (8.3%), Ghana, and Nigeria (7.2% each). Sixty-two percent of travelers visited malaria endemic areas.

Table 1 describes travelers' and travel characteristics. The majority of international travelers were male, of Greek nationality and with tertiary education. One-third of the travelers were foreign born; 56.1% of them were born in North Africa (mainly in Egypt) and Ethiopia. Outdoor activities and contact with animals were reported by 67 (18.6%) and 15 (4.2%), respectively. Those traveling for leisure and VFRs were more likely to be involved in outdoor activities than those traveling for work (31.9% and 29.9%, resp., versus 15.4%) (*P* value < 0.001).

Half of all travelers reported having sought pretravel consultation and most of them sought consultation 15–28 days prior to departure (Table 1). The majority of those who pursued health information (61.5%) had a history of previous travel to a tropical country (*P* value < 0.001), traveled to sub-Saharan Africa (89.4%) (*P* value < 0.001), and stayed in local houses (56.1%) (*P* value < 0.001). Of those traveling for business, leisure, and VFRs, 60%, 24.4%, and 12.2% sought pretravel advice, respectively (*P* value < 0.001).

Vaccination (previous vaccination or vaccination prior to the current trip) according to destination is described in Table 2. A total of 572 vaccines were administered to 136 (37.8%) of travelers (mean number of vaccines: 4.2, range: 1–14); 122 (89.7%) and 14 (10.3%) received travel and routine vaccines, respectively. The vaccines most commonly administered were hepatitis A, yellow fever, and tetanus/diphtheria vaccines. More than 90% of travelers visited areas of high endemicity for hepatitis A, and nearly 80% of them areas endemic for typhoid fever. Hepatitis A and typhoid vaccination rates were lower than expected (60% and 55%, resp.). All travelers who received meningococcal vaccine traveled to sub-Saharan Africa (16.1%); retrospective analysis of data based on travelers' and travel characteristics, revealed that this rate was lower as expected by 25%. Similarly, all of those to whom yellow fever vaccine was administered traveled to sub-Saharan Africa; this rate was considered slightly lower than expected (10%). Rabies vaccine was administered to only 1.9% of all travelers and only to travelers visiting sub-Saharan Africa.

Vaccination in relation to purpose of travel revealed that more of those who traveled for recreation received hepatitis A vaccine than business travelers, VFRs, and tourists (54.2% versus 47.5% and 5.8%, resp.) (*P* value < 0.001), whereas more business travelers were administered typhoid fever vaccine than VFRs and tourists (40.6% versus 2.2%, 0%, resp.) (*P* value < 0.001). Similarly, more business travelers received meningococcal vaccine than tourists and VFRs (32.2% versus 11.1% and 1.5%, resp.) (*P* value < 0.001). Yellow fever was delivered to 44.8% of business travelers, 37.5% of tourists, and 6.6% of VFRs (*P* value < 0.001).

The association between tetanus/diphtheria, poliomyelitis, hepatitis B, hepatitis A, typhoid, yellow fever and meningococcal vaccination, and destination and more specifically with sub-Saharan Africa was statistically significant (*P* value < 0.001).

Malaria prophylaxis according to destination is shown on Table 3. A total of 146 travelers were prescribed chemoprophylaxis. Mefloquine was the most commonly prescribed antimalarial (54.7%). Nearly 22% (77) of travelers who visited malaria endemic areas did not receive any antimalarials.

There was a statistically significant association between malaria chemoprophylaxis, general advice, and destination. Most of those who traveled to sub-Saharan Africa (66.8%) were prescribed chemoprophylaxis than to other destinations (*P* value < 0.001); 55.2% and 44% of them received mefloquine and atovaquone/proguanil, respectively. In terms of purpose of travel, more business travelers (66.9%) received antimalarials than tourists (51.4%) and VFRs (6.6%) (*P* value < 0.001).

Table 4 describes travelers' and travel characteristics in relation to purpose of travel. The association of purpose of travel (recreation, work, and VFRs) with age, gender, education, employment, destination, place and area of stay, and duration of travel was statistically significant (*P* value < 0.001).

Multivariate analysis was used in order to investigate the association of several factors with failing to pursue pretravel health consultation among travelers to African destinations. Having a history of previous travel to a tropical country, having an elementary or secondary level of education, traveling for visiting friends and relatives, *k* traveling for short duration and traveling with someone were significant determinants for not pursuing pretravel consultation (Table 5).

## 4. Discussion

During the period 2009-2010 nearly 34,000 travelers from Greece visited countries of Africa [18]. Most of these destinations are endemic to a variety of infectious diseases which may cause considerable morbidity and mortality. The aim of the current survey is to study the profile of Greek travelers visiting Africa, and their pretravel health seeking practices including vaccine and malaria prevention.

The profile of the participants in the present study revealed that more than two-thirds of international travelers were travelers with employment and a history of previous travel to tropical and subtropical countries and with tertiary level of education. Travelers with this profile are expected to be more receptive to awareness about health prevention. However, the overall level of practice regarding vaccinations and malaria chemoprophylaxis was low.

In regard to the demographics of the study population the results were comparable to those of other similar studies; most of the travelers were men and young or middle aged [7–12]. The percentage of those traveling alone was higher compared to that of other studies [8, 10]. Nearly thirty percent traveled to tropical and subtropical countries for the first time. Similar rate was noted in a Spanish study [8].

In regard to purpose of travel, the results of the present survey showed that the number of those traveling for the purpose of work or VFRs was higher, whereas the rate of those traveling for leisure was lower compared to the results of other studies [7, 8, 10, 11]. VFRs traveled predominantly to North Africa, which may reflect the composition of the African VFR population in Greece, and those who traveled for recreation and work visited mainly sub-Saharan Africa. The majority of business travelers stayed for long period of time, which explains the fact that most of them stayed in local houses. Compared to a previous Greek study the current survey shows a changing profile of Greek travelers to developing countries [19]; this may be due to the influence of economic crisis in Greece which is reflected on immigration and international travel.

In the current study, less than half of the travelers reported having sought pretravel consultation; similar rates were found in other studies [7–13]. VFRs pursued less often health information than those traveling for leisure or for work. Inadequate pretravel preparation, visiting rural areas, traveling for longer periods of time, and staying with local people in developing countries predispose VFRs, compared with other travelers, to many largely preventable infectious diseases [3–6, 20].

Vaccination rate in the current survey was low; however it was comparable with the results of other studies [8, 10–12]. Hepatitis A vaccine was the prevalent vaccine delivered. Similarly, this was the most common vaccine administered in similar studies [8, 10–12]. Although more than 90% of travelers visited areas endemic for hepatitis A, vaccination rate for hepatitis A was lower than expected as shown by retrospective analysis of travel and travelers' characteristics. A possible reason may be previous vaccination as part of the National Immunization Program of Greece or previous travel to tropical or subtropical countries. However, lack of knowledge about risk destinations may be another reason [21, 22].

Typhoid vaccine was delivered to 17.5% of travelers; this rate was lower compared to the results of other studies [8, 12]. Nearly 80% of all travelers visited typhoid fever endemic countries. Although VFRs accounted for more than one-third of the travelers and more than 80% of them stayed for ≥1 month and nearly all of them at local houses, only a few of them (<2%) received typhoid fever vaccine; this may be the result of inadequate pretravel health seeking practices of VFRs as shown by a previous Greek study [20]. Retrospective analysis based on travel and travelers' characteristics showed that the vaccination rate against typhoid fever was lower than expected (55%).

Meningococcal vaccine was administered to those traveling to sub-Saharan Africa. Nearly two-thirds of the travelers to sub-Saharan Africa intended to stay for long period of time and for work; as shown by retrospective analysis of data this rate was lower by 25%. Meningococcal vaccination should be considered in “all travelers to countries in the sub-Saharan meningitis belt” in particular for those traveling during the dry season or those staying in the area for longer periods and living with or being in close contact with the local population for whom the risk of infection may be greater [23, 24]. Yellow fever vaccine was administered only to travelers visiting sub-Saharan Africa (47.9%); lower vaccination rate was justified due to previous immunity as shown by retrospective analysis.

Rabies vaccine was administered to only 1.9% of all travelers and only to travelers visiting sub-Saharan Africa (3.3%). Only 0.1% of travelers VFRs and 0.6% of long-term travelers were vaccinated against rabies despite traveling to a rabies-endemic country. Travelers' and travel health providers' lack of knowledge about the risks of rabies exposure may be related to the low vaccination rates against rabies, as shown in recent studies [25, 26]; results from a previous Greek survey showed that travel health consultants' knowledge regarding preexposure rabies prophylaxis was deficient [25].

In the current study, vaccination with routine vaccines such as tetanus/diphtheria was comparable to the rates of another study [10]. Previous vaccination coverage through the National Vaccination Program of Greece which is provided by health professionals other than travel health consultants may be responsible for low vaccination rates with routine vaccines [27]. Since influenza is considered as the most common travel-associated, vaccine-preventable infectious disease it is important to increase travelers' awareness of influenza risk distribution and prevention measures [28]. Furthermore specific recommendations are needed which address the indications for influenza vaccination in travelers [29].

Malaria risk was assessed according to destination countries, area and place of stay, purpose, and duration of travel. The rate of malaria chemoprophylaxis in the current study was comparable to the rate in other studies [8–10, 12, 30]. None of the travelers was prescribed SBET (Stand By Emergency Treatment). Retrospective analysis of data based on travel and travelers' showed that approximately 22% of all travelers who visited malaria endemic areas did not receive any antimalarials. Only 6.6% of VFRs and 67% of business travelers who traveled to malaria endemic areas were prescribed chemoprophylaxis, whereas approximately 8% of business travelers who traveled for more than six months to malaria endemic areas had no information about SBET; for travelers visiting high risk areas and staying in remote locations for prolonged period of time SBET is indicated [15]. These results suggest that malaria prophylaxis was inadequate, in particular with regard to special groups, such as VFRs and business travelers which may be related to their lack awareness about the risk of the disease [22, 30].

## 5. Conclusions

Travel medicine providers were underutilized with only one half of all travelers to African destinations reporting having sought pretravel consultation. Inadequate vaccination coverage and malaria prevention of travelers to high risk destinations suggest that public health strategies should be developed in order to increase travelers' awareness about the risk of exposure to travel-related infectious diseases and the importance of pretravel consultation. In particular, there is a special need for education initiatives to target high risk groups of travelers such as travelers VFRs and business travelers. Travel health providers should increase their efforts through training and continuing professional development in order to propagate safe and healthy travel.

## Figures and Tables

**Table 1:** Characteristics of travelers to African countries and pretravel health advice (*N* = 360).

	*N* (%)
Gender	
Male	245 (68.1)
Age (years)	
19–34	106 (29.4)
35–49	215 (59.7)
50–64	33 (9.2)
≥65	6 (1.7)
Education	
University/tertiary	235 (65.3)
Secondary school	51 (14.2)
Elementary school	67 (18.6)
Nationality	
Greek	230 (63.9)
Other	130 (36.1)
Employment	
Yes	314 (87.2)
Previous travel to tropical or subtropical country	
Yes	259 (71.9)
Traveled with	
Alone	200 (55.6)
Friends and relatives	61 (16.9)
Husband/wife	46 (12.8)
Group	24 (6.7)
Children	28 (7.8)
Other	1 (0.3)
Duration of travel	
<1 month	137 (38.1)
1–3 months	117 (32.5)
3–6 months	49 (13.6)
>6 months	57 (15.8)
Purpose	
Recreation	72 (20)
Work	143 (39.7)
VFR's	137 (38.1)
Religious reasons	1 (0.3)
Education	5 (1.4)
Other	2 (0.6)
Place of stay	
Local residence	230 (63.9)
Hotel	114 (31.7)
Ship	9 (2.5)
Camping	6 (1.7)
Other	1 (0.3)
Area of stay	
Urban	233 (64.7)
Urban and rural	89 (24.7)
Rural	35 (9.7)
Time in which pretravel health advice was sought before the journey (d)	
Yes	180 (50)
≤7	11 (3.1)
8–14	62 (17.2)
15–28	66 (18.3)
≥28	41 (11.4)
Sources of pretravel advice	
Yes	170 (47.2)
Public Health Department	153 (42.5)
HCDCP	103 (28.6)
Internet	34 (9.4)
Primary Care Doctor	31 (8.6)
Other	4 (1.1)
Hospital	0 (0)

**Table 2:** Vaccine prophylaxis according to destination*.

	*N* (%)		
	Sub-Saharan Africa	North Africa	South Africa
	*N* = 216	*N* = 126	*N* = 18
Routine vaccines**			
Hepatitis A	108 (49.8)	8 (6.4)	2 (11.1)
Tetanus/diphtheria	95 (43.8)	6 (4.8)	2 (11.1)
Hepatitis B	36 (16.3)	2 (1.6)	0 (0)
Poliomyelitis	39 (18.1)	0 (0)	0 (0)
Measles, Mumps, Rubella	13 (6.1)	0 (0)	0 (0)
Influenza	7 (3.3)	1 (0.8)	0 (0)
Pneumococcal	5 (2.3)	0 (0)	0 (0)
Travel vaccines			
Typhoid fever	61 (28)	2 (1.6)	0 (0)
Cholera	6 (2.8)	0 (0)	0 (0)
Yellow fever	104 (48)	0 (0)	0 (0)
Meningococcal (A, C, W135, Y)	58 (26.6)	0 (0)	0 (0)
Rabies	7 (3.3)	0 (0)	0 (0)
Tick-born*** encephalitis	5 (2.3)	0 (0)	0 (0)
Japanese encephalitis****	5 (2.3)	0 (0)	0 (0)

*Vaccination was administered to 136 travelers (37.8%).

**In accordance with the National Vaccination Program of Greece.

***Recent vaccination for a trip to tick-born encephalitis endemic countries.

****It was administered to travelers with South East Asia as a second destination.

**Table 3:** Malaria chemoprophylaxis according to destination*.

	Mefloquine	Atovaquone/proguanil	Doxycycline	Total
North Africa	0 (0)	0 (0)	0 (0)	126
Sub-Saharan Africa	79 (36.7)	63 (29.3)	1 (0.5)	216
South Africa	1 (0)	2 (11.8)	0 (0)	18
Total	**80 (22.2)**	**65 (18)**	**1 (0.3) **	**360**

*Malaria chemoprophylaxis was prescribed to 146 travelers (40.6%).

**Table 4:** Travelers and travel characteristics by purpose of travel.

	Tourists	Business travelers	VFRs	*P* value
Age				<0.001
19–34	39 (54.2)	24 (16.8)	39 (28.5)	
35–49	25 (34.7)	103 (72)	83 (60.6)	
50–64	8 (11.1)	13 (9.1)	12 (8.8)	
≥65	0 (0)	3 (2.1)	3 (2.2)	
Gender				<0.001
Male	25 (34.7)	126 (88.1)	90 (65.7)	
Nationality				
Greek	69 (95.8)	140 (97.9)	13 (9.5)∗	<0.001
Other	3 (4.2)	3 (2.1)	124 (90.5)	
Education				
University/tertiary	63 (87.5)	132 (92.3)	32 (24.6)	<0.001
Secondary school	7 (9.7)	7 (4.9)	37 (28.5)	
Elementary school	2 (2.8)	4 (2.8)	61 (46.9)	
Employment				<0.001
Yes	69 (95.8)	142 (100)	96 (70.1)	
Duration of travel				<0.001
<month	68 (94.4)	44 (30.8)	21 (15.3)	
1–3 months	2 (2.8)	37 (25.9)	75 (54.7)	
3–6 months	2 (2.8)	20 (14)	27 (19.7)	
>6 months	0 (0)	42 (29.4)	14 (10.2)	
Destination				<0.001
North Africa	22 (30.6)	25 (17.5)	77 (56.2)	
Sub-Saharan Africa	44 (61.1)	111 (77.6)	55 (40.2)	
South Africa	6 (8.3)	7 (4.9)	5 (3.7)	
Place of stay				<0.001
Hotel	68 (94.4)	44 (30.8)	1 (0.7)	
House	1 (1.4)	87 (60.8)	136 (99.3)	
Camping	3 (4.2)	3 (2.1)	0 (0)	
Ship	0 (0)	9 (6.3)	0 (0)	
Area of stay				<0.001
Urban	64 (88.9)	112 (80)	52 (38)	
Urban + rural	6 (8.3)	21 (15)	59 (43.1)	
Rural	2 (2.8)	7 (5)	26 (19)	

*People of African origin with Greek citizenship.

**Table 5:** Multivariate analysis of factors associated with not pursuing health information among travelers to African countries.

Characteristics	OR (95% CI)	*P* value
Education level		
Elementary school	10.2 (3.58–29.22)	<0.001
Secondary school	4.89 (2.10–11.42)	<0.001
Purpose of travel		
VFRs (visiting friends and relatives)	5.23 (2.46–11.11)	<0.001
Duration of travel		
<1 month	3.97 (2.05–7.67)	<0.001
Traveling with someone		
Friends and relatives	2.66 (1.20–5.88)	0.016
Previous travel		
Yes	2.86 (1.49–5.48)	0.002
